# Residual Stress Evaluation in L-PBF-Produced SS 316L Specimens

**DOI:** 10.3390/ma17102270

**Published:** 2024-05-11

**Authors:** Matúš Geľatko, Michal Hatala, František Botko, Radoslav Vandžura

**Affiliations:** Faculty of Manufacturing Technologies, Technical University of Košice with a Seat in Prešov, 080 01 Prešov, Slovakia; matus.gelatko@tuke.sk (M.G.);

**Keywords:** additive manufacturing, selective laser melting, non-destructive testing, X-ray diffraction, stainless steel

## Abstract

The identification of residual stresses (RS) in components made by selective laser melting (SLM) is necessary for subsequent technological optimization. The presented research is devoted to evaluating the influence of the combination of laser power (P), scanning velocity (v) and the rarely considered number of layers (n_L_) on surface residual stresses in SLM stainless steel SS 316L. Experimental parameters were set based on the Design of Experiment (DoE) method, with follow-up X-ray diffraction (XRD) measurements and data processing using analysis of variance (ANOVA) and regression analysis. The obtained data are a valuable stepping-stone for the subsequent design of research focused on the application of sustainable eco-friendly Abrasive Water Jet (AWJ) peening for RS modification in the evaluated material.

## 1. Introduction

The surface integrity of materials is an important discipline that expresses their state from a qualitative point of view, including certain parameters that define its level. Stress is one of the most influential elements that participates at different phases of all manufacturing processes. The character and quantity of stress varies during individual operations and remains in the final component on non-zero values as residual stresses that can be compressive, which are normally positive, and tensile, which are normally considered to be negative, mainly due to their supportive character on the growth of discontinuities. Three main actuating mechanisms are the thermal phase transformation mechanism, thermal-plastic deformation mechanism and mechanical deformation mechanism. The first is commonly represented by sharp thermal gradients that cause material volume variations, e.g., during heat treatment. The second is commonly represented by the combination of mechanical and thermal load, which occurs during some machining technologies. The third is represented by the dominating mechanical load during technologies such as polishing or shot peening [1,2].

The SLM process is characterized by the input material in the form of powder, which is transformed into the solid state using the laser beam with high power impact. The powder becomes fully molten, is subsequently cooled and the final component is created by the layer-by-layer repetition of this operation. Considering that the material is subjected to the laser impact, which focuses its high energy on the one small point during the SLM process, actuating mechanisms of residual stress induction can be predicted in the form of sharp thermal gradients around this point. The material is not able to absorb this heat, the expanding surface layer is restricted by subsurface layers and compressive residual stresses arise. After reaching the yield strength of the material, plastic deformation of the surface layer occurs, which consequently leads to its shrinking during cooling, whereas this phenomenon is again restricted by subsurface layers; hence, the final residual stresses in the surface layer are of tensile character. Applying other surface layers causes the reduction of tensile stresses in the subsurface layers. In the final SLM component, maximal tensile residual stresses are present on the surface with a decrease of their value deeper into the volume [3,4,5]. A variation in the stress state is influenced by factors included in Table 1.

**Table 1:** Factors influencing residual stresses in SLM components.

Tensile Residual Stresses	Factor
Increasing	Number of layers [3]
	Material yield strength [3]
	Overall energy input [5,6]
	Laser power [7,8]
	Scanning length [5]
Decreasing	Substrate thickness [3]
	Scanning velocity [5,7]
	Layer thickness [9]
	Scanning strategy [10,11]
	Re-scanning [12]
	Substrate preheating [11,12]

It is evident that several factors influence the induction of residual stresses during AM, but the key is the energy density that causes the variation in residual stresses, which is described within the experiment focused on the AM material AISI 316L [6]. Chiefly, the laser power and scanning velocity are the most crucial at this energy density. As was stated in one study [7], the most important factor can be defined as the main mechanism of energy input and the second most important can be defined as the main mechanism of cooling rate. A more significant induction of residual stresses occurs with increasing laser power, which can be considered a negative factor [8]. In contrast, the second factor can be considered the positive effect, due to the shorter duration of laser energy impact on the influenced area of the material with increasing of scanning velocity [5]. Authors Mercelis and Kruth [3] described the increasing residual stresses caused by the greater number of applied layers.

Considering the previously mentioned influence of residual stresses in the surface layers of the materials, X-ray diffraction non-destructive testing seems to be adequate for their measurement [13]. This was confirmed by recent studies focused on RS evaluation, where X-ray diffraction was applied to a wide portfolio of SLM materials. On the Al-12 Si aluminum alloy, XRD was used to record differences in residual stresses after the heat treatment at 540 °C for 1, 7 and 26 h [14]. Titanium alloy Ti6Al4V was the material of interest in the comprehensive study of the effect of selected post-processing technologies on basic material properties, where XRD took part in the evaluation of residual stresses [15]. During the monitoring of RS formation in Inconel 625 alloy, synchrotron XRD was used for the in situ measurement with promising results [16]. The effect of build location during the L-PBF technology on roughness and residual stresses (using XRD) in Maraging 300 steel was evaluated during the experiment performed by de Oliveira and co-authors [17]. Specimens of SLM 316L stainless steel were measured using X-ray diffraction during the experimental validation process of FEM analysis focused on the thermal and residual stress profile in identical material [18]. Chao et al. used the XRD method as a tool to monitor the influence of post-processing heat treatment (400–1400 °C) on residual stress relief and other properties of SLM 316L material [19]. Preheating and re-scanning techniques can similarly influence the behavior of residual stresses in SLM stainless steel, which was investigated using FEM analysis with XRD validation [12].

The design of experiment (DoE) method allows the statistical analysis of the effect of various technological parameters on monitored output parameters, not excluding residual stresses, such as those carried out within the experiment focused on GTAW (Gas Tungsten Arc Welding) [20], or the optimization of the SLM process parameters´ influence on final porosity, experimentally conducted on AZ31 magnesium powder [21]. Such statistically oriented studies include the follow-up application of ANOVA (analysis of variance) [22] and regression analysis [23] for the expression of evaluated dependencies. Considering that various analytical methods are crucial within the evaluation of processes [24], a combination of previously mentioned statistical methods with XRD non-destructive testing could be an appropriate tool for the prediction of induced surface residual stresses in SLM stainless steel based on the magnitude of selected technological parameters.

The presented experiment is focused on the evaluation of selected SLM parameters, considering the final surface residual stresses in SLM stainless steel with the application of methods, mentioned in the previous paragraph. The motivation for the research was to monitor the influence of selected parameters´ combinations on final surface residual stresses, mainly due to the absence of experimental data related to the number of layers’ influence and follow-up SLM process optimization. Various RS values in experimental specimens, obtained by the application of the DoE method, will be crucial for the subsequent setting of AWJ peening application, within research focused on their reduction.

## 2. Materials and Methods

The research was conducted using a Design of Experiment (DoE) method within the Minitab v21 statistical software, which allows the monitoring of the output data based on the input data, with the expression of their mutual relations and significance, whereas the output data (responses: y_1_, y_2_… y_n_) are dependent on the input data (predictors: x_1_, x_2_… x_m_), which are expressed by values defined as factorial levels. The number of runs for a certain number of predictors (factors) and their levels can be expressed by Equation (1) [25]:

n = λ^k^ (1)

where λ represents the number of factorial levels (inputs) and k represents the number of factors. The primary objective is a definition of a mathematical model, expressing previously mentioned relations and the secondary objective is their subsequent analysis and graphical and numerical interpretation. The mathematical model, important for the optimization and subsequent experiments, can be expressed by a polynomial function, which corresponds to Equation (2) [25]:

y_i_ = b_0_ + b_1_ × x_1_ + b_2_ × x_2_ + b_3_ × x_3_ + b_12_ × x_1_ × x_2_ + b_13_ × x_1_ × x_3_ + b_23_ × x_2_ × x_3_ + b_123_ × x_1_ × x_2_ × x_3_ (2)

where y_i_ is the observed value of output quantity y (response), x_i1_…x_ik_ are values of input quantities (factors), b_0_ is the average value (intercept), and b_i_1…b_ik_ are coefficients of regression equation [25]. For the experiment, three factors were selected in the form of laser power (P), scanning velocity (v) and number of layers (n_L_). For the response, resulting residual stresses σ were monitored on the surface layer of experimental specimens. The influence of the mentioned factors was analyzed at two levels with two replicates, which corresponds to the two-level factorial design of experiment 2^3^ with an overall number of 16 repetitions. Therefore, 16 experimental specimens were made of SS 316L stainless steel (Table 2 and Table 3) using the SLM technology, during which the powder material is made fully molten by the laser energy impact, and after its cooling, a homogenous structure is created. Specimens were prepared at the Technical University in Ostrava (Czech Republic) Protolab Center for 3D printing on a RenAM500S Flex machine by Renishaw company (Wotton-under-Edge, UK).

Table 4 includes technological parameters of the experimental specimens’ creation. Boundary values of parameters (P, v and n_L_), representing factors within the two-level model, were set based on their influence on the porosity formation, which was monitored within the recent research [27]. The reason was the evaluation of residual stresses in possibly the most homogenous material.

**Table 4:** SLM process parameters.

Parameter	Symbol	Value	Unit
Laser power	P	200–300	W
Scanning velocity	v	500–800	mm·s^−1^
Hatching distance	d	110	μm
Layer thickness	t_L_	50	μm
Number of layers	n_L_	200–400	-
Scanning strategy		Meander (Figure 1)	
Gas protection		Argon (Ar)	

**Figure 2:**
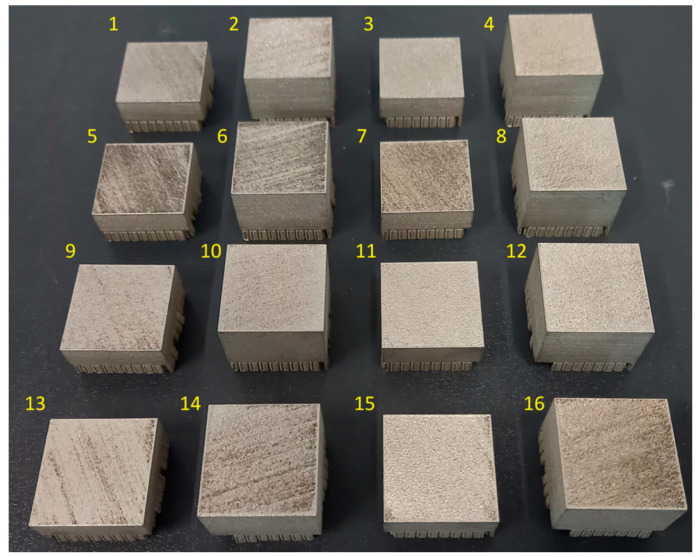
Experimental specimens made of SLM SS 316L material (according to DoE).

Specimens of cut square (Figure 2) were designed and prepared in dimensions 30 × 30 mm and heights of 10 and 20 mm, based on the number of applied layers (200 and 400). A larger size of experimental specimens was selected with consideration to the conduction of subsequent experiments, focused on the application of Abrasive Water Jet (AWJ) peening. Table 5 includes values of selected SLM parameters for each specimen.

The X-ray diffraction (XRD) method was used for the evaluation of residual stresses in the surface layer of experimental specimens. Measurements were performed at the Department of Machining and Manufacturing Technologies at the University of Žilina on Proto iXRD machine with an MG40 goniometer by Proto company (Wrocław, Poland), using the parameters summarized in Table 6. Overall, 80 measurements were performed on 16 specimens.

**Table 6:** X-ray diffraction parameters.

Parameter	Value	Unit
X-ray tube	Mn_K (α)	-
Filter	Cr	-
Collimator diameter	1	mm
Voltage	20	kV
Current	4	mA
Beta oscillation (β)	3	°
Number of angle positions	15 (±30°)	-
Penetration depth	10	μm
Number of points on specimen	5 (Figure 3)	-

## 3. Results

Obtained values of normal surface residual stresses at five points on each specimen were averaged and these mean values were used as fundamentals for the subsequent evaluation of influencing factors in the form of selected technological parameters (Table 5).

**Figure 4:**
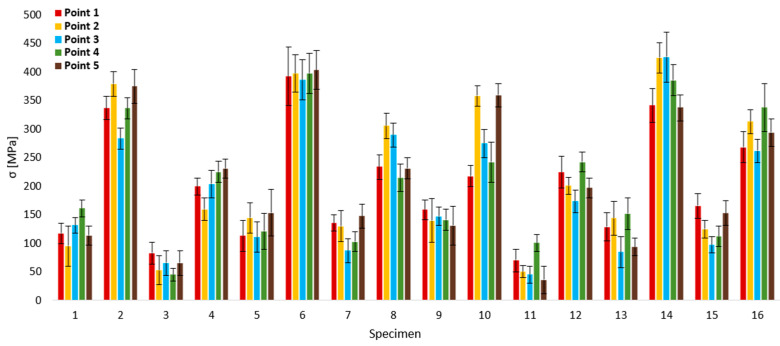
Residual stresses in individual points of specimens (XRD observed).

As could be assumed, the residual stresses in the surface layers of all specimens, measured at all points, were of tensile character, which is characteristic for the residual stress induction mechanism caused by the impact of a laser beam with high energy density during SLM. The next diagram (Figure 4) allows the interpretation of values of residual stresses in individual points of specimens with included variances, representing a distribution uniformity of residual stresses in measured points, whereas a lower value indicates a higher uniformity. The lowest value of mean residual stresses was reached in the case of specimen 11 (60.28 MPa), which can be ascribed mainly to the influence of a higher scanning velocity v (800 mm∙s^−1^) in combination with a lower laser beam power P (200 W). In contrast, the residual stresses with the highest mean value were present in specimen 6 at 395.1 MPa. The primary factor of such an elevated value can be predicted mainly as the influence of number of layers n_L_ (400) and scanning velocity v on the value of 500 mm∙s^−1^.

The lowest value of variance was reached at point 2 within the measurement of specimen 11, on the level 10.9 MPa. Inversely, the highest variance was reached at point 1 of specimen 6, with the value 51.2 MPa. Considering all measurements of residual stresses, the lowest value was in point 5 of specimen 11, equal to 35.9 MPa, and vice versa, the highest value of residual stress was present at point 3 of specimen 14, on level 425.6 MPa. A difference between the minimal and maximal values of residual stresses within individual specimens can be considered the indicator of their distribution in the area of five measurement points. Similarly to the variance, the lower the value, the more uniform the residual stress in the area of five measurement points. The mentioned difference was calculated with the lowest value within specimen 6 and with the highest value within specimen 10, equal to 17.5 MPa and 141.8 MPa, respectively.

**Figure 5:**
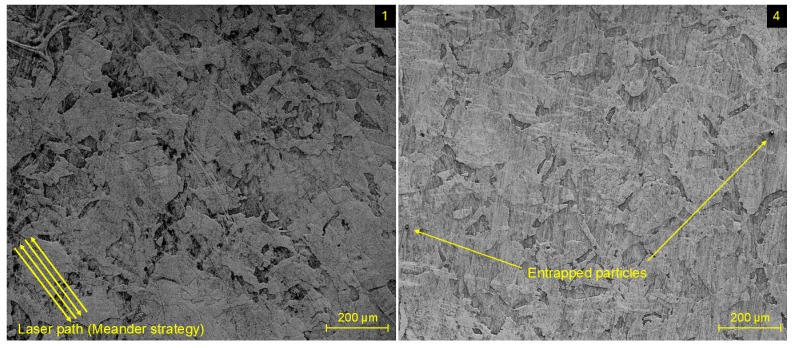
Microstructure of experimental specimens 1 and 4.

To check the correctness of setting the limit values of the SLM parameters and their influence on the homogeneity of the microstructure, SEM analysis was performed on the surface layer of specimens within one replication (specimens 1–8). The evaluated specimens are composed of an austenitic microstructure, characteristic of low-carbon stainless steels. In comparison with conventional AISI 316L stainless steel, where the grains are equiaxed, the SLM process caused the creation of a typical LPB-F microstructure in the form of grains of irregular shapes and orientations. Certain grains tend to elongate in the direction of the path of the laser beam within the meander scanning strategy, which is depicted in a diagonal direction on the surface of specimen 1 in Figure 5.

**Figure 6:**
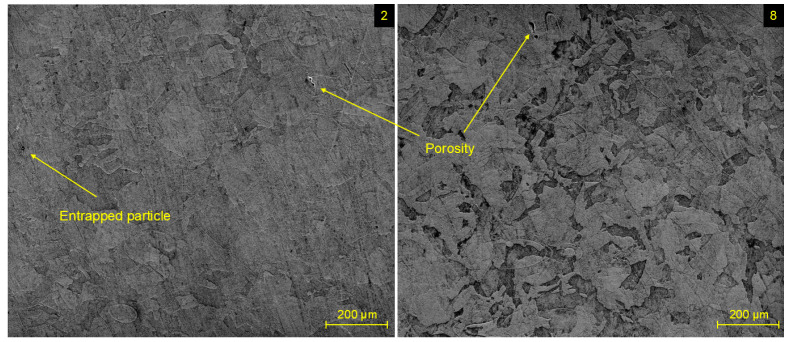
Microstructure of experimental specimens 2 and 8 with entrapped particles and porosity.

SEM image of specimen 4 (Figure 5) revealed the presence of powder particles entrapped within its microstructure. Such irregularities are common during all kinds of L-PBF processes, where sporadic insufficient melting of individual particles can occur. The presence of such single particles was observed in a negligible number within all evaluated specimens. In addition to the entrapped particle, Figure 6 depicts the occurrence of porosity in a microstructure of specimen 2 and specimen 8. Such types of voids, as the consequence of gas entrapment in the local melt pool during the cooling process, were not recorded in a systematic order and similarly were present in a negligible number.

**Figure 7:**
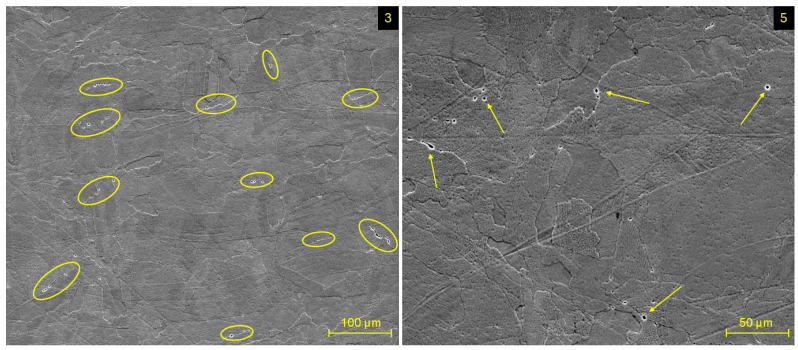
Inclusion on grain boundaries in specimens 3 and 5.

At a higher magnification, inclusions on the grain interfaces were recorded for all evaluated specimens (Figure 7). Such discontinuities were present in the form of clusters containing the set of smaller inclusions and also in the form of individual inclusions of bigger size. A potential explanation for their presence can be a δ-ferrite, as the consequence of the precipitation process at numerous preheating temperatures, which take place during the SLM process when a heat source influences the material multiple times throughout the transition of a laser beam. The elevated percentage of Cr and Mo elements in material composition supports the creation of this phase, due to their ferrite-forming nature [29].

**Figure 8:**
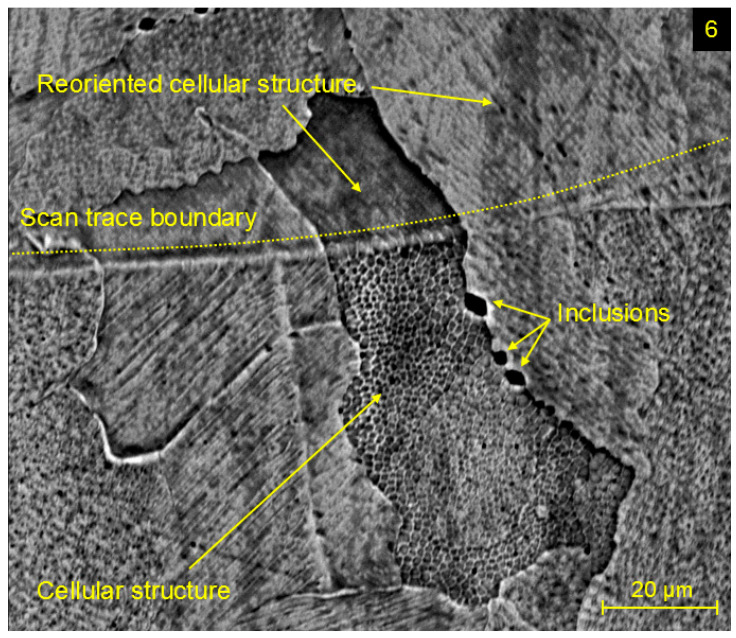
SEM image of grain interface in the microstructure of experimental specimen 6.

Figure 8 includes an SEM image of the microstructure at the level of the grains, and a size of 10–50 μm was observed for all experimental specimens. These austenitic grains are composed of a fine cellular structure, which arises during the SLM process, mainly as a consequence of the rapid cooling of molten material. The cellular structure is variously oriented within grains, whereas its reorientation during the change of morphology arises without some external mechanism in the areas of grain boundaries, scan trace boundaries, or melt pool interfaces. Also, previously described inclusions on grain boundaries were observed in Figure 8.

It can be stated that the SEM observed data confirmed the correctness of setting the limit values of SLM parameters, considering the integrity of the microstructure. Observed discontinuities in selected specimens were shown to be obvious for the manufacturing process used for the material (SLM)—sporadic entrapped powder particles and precipitation inclusions on grain boundaries, or voids in the form of gas-entrapment porosity that were identified randomly, in negligible number and smaller sizes. The influence of such discontinuities on the RS character, their magnitude and other mechanical properties can be considered insignificant; thus, the RS can be evaluated only with respect to the SLM parameters.

### 3.1. Analysis of Variance (ANOVA)

The mean values of residual stresses were used for the creation of analysis of residual stresses on the surface layer of the experimental material. The selected factors (P, v and n_L_) were evaluated in terms of their influence on the residual stress response (σ), using the analysis of variance method (ANOVA), based on which their statistical significance can be assessed, where the key parameter is the *p*-value (Table 7). For the assessment of statistical significance, the *p*-value is considered with respect to the level of significance α = 0.05. Considering that the mentioned value did not exceed this level at any factor, the null hypothesis H_0_ can be accepted and, consequently, the individual factors were regarded as statistically significant. The calculated coefficient of determination R^2^ is 0.9610, which means that the model can be applied in 96.10% of similar cases, as described by Equation (3). The statistical significance of the described model is also confirmed by the adjusted coefficient of determination R^2^_Adj_ with a value of 0.9468 at the 94.68% level.

### 3.2. Regression Analysis

As previously mentioned, Equation (3) expresses the residual stresses (σ) induced in the surface layer of the experimental material, as the response to the influence of selected factors in the form of laser power (P), scanning velocity (v) and number of layers (n_L_).

σ = − 286.6 + 0.493 × P + 0.137 × v + 175.9 × n_L_ − 0.001280 × v × n_L_ (3)

**Figure 9:**
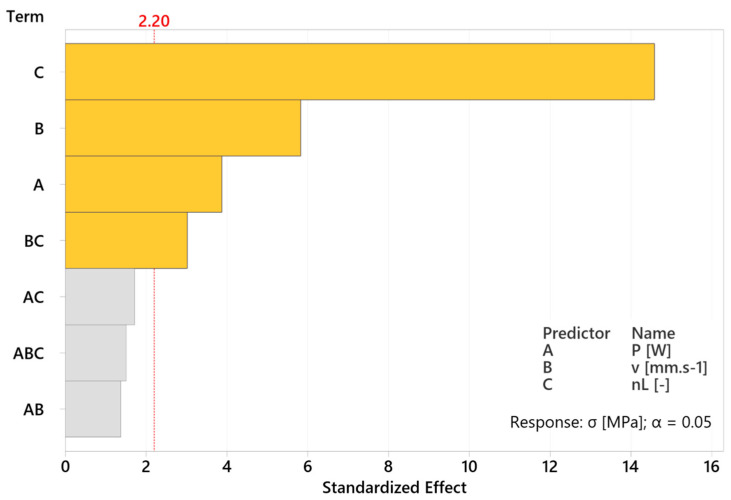
Pareto chart of standardized effects (P, v, n_L_ and their interactions) on RS.

The Pareto chart of the standardized effects (Figure 9) shows an interpretation of the result of individual factors’ significance analysis in the form of technological parameters, including their combinations for the subsequent creation of a regression model. The most influential factor with a significantly higher value was shown to be factor C, which corresponds to the number of layers n_L_ on the value 14.6. The second most significant influence had factor B, which represents the scanning velocity v and the factor with the least significance was factor A, representing the power of laser beam P, on the values 5.83 and 3.88, respectively. In addition to the mentioned three individual factors, the BC factor corresponding to the combination of scanning velocity v and a number of layers n_L_ (3.02) is present above the limit of the critical value of standardized effect (2.20). All these factors are important for the creation of a regression model. Reversibly, other 2 and 3-factorial combinations (AC—1.72, AB—1.51, ABC—1.37) are below the limit of critical value; hence, they are negligible for the creation of a regression model.

**Figure 10:**
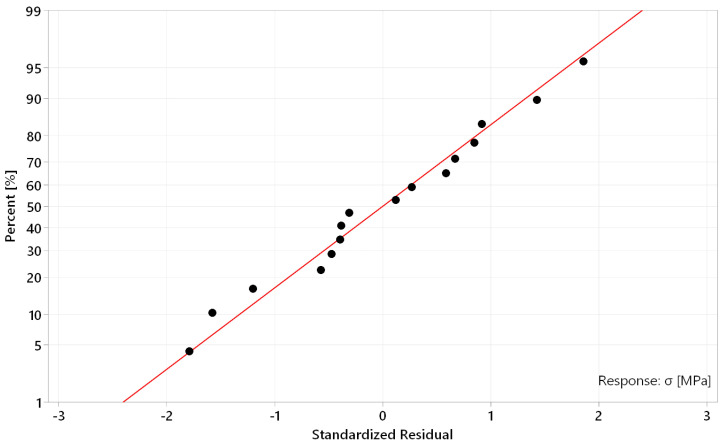
Normal probability plot of standardized residual distribution.

A normal probability plot of residual distribution, within a described regression model, is shown in Figure 10. It can be stated that the distribution of residuals acquires a state of normality, taking the positions of individual points, which are situated in the close area of the curve, representing the ideal state, with minimal deviations. Any of included points did not exceed value 2 of the Standardized Residual and most of them are present in the range of the Standardized Residual value 1 in both directions, which confirms the correctness of the regression model.

**Figure 11:**
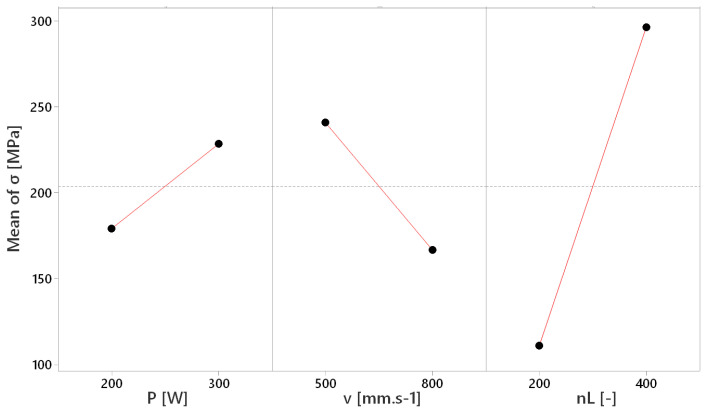
Plot of main effects (P, v and n_L_) on residual stresses (σ).

The main effects plot, included in Figure 11, puts selected individual factors (P, v and n_L_) and values of residual stresses (σ) into proportion, by which the influence of independent variables on a dependent variable within the regression model are interpreted. The reference line is on 203.7 MPa. It is evident that the least significant influence on the induction of residual stresses is present in the given range of laser power P values. However, this parameter needs to be taken into account, due to the increase in induced residual stresses with its increase, which can be considered a negative influence. The magnitude of residual stresses varies from 179.1 MPa at 200 W to a maximum of 228.3 MPa at 300 W. The scanning velocity (v) influences the formation of residual stresses in a greater manner, but it needs to be pointed out that this influence is positive, because the residual stress value decreases with increases in velocity, due to the shorter time of laser power impact into the single point. The maximum was reached at 500 mm.s^−1^ at 240.7 MPa and decreased to 166.7 MPa at 800 mm.s^−1^. The most significant influence was recorded at the number of layers (n_L_), when a radical graduation of tensile residual stress component occurs with the increasing of layers. The overall maximum of all factors was reached at 296.3 MPa at 400 layers to the smallest value 111 MPa at 200 layers.

**Figure 12:**
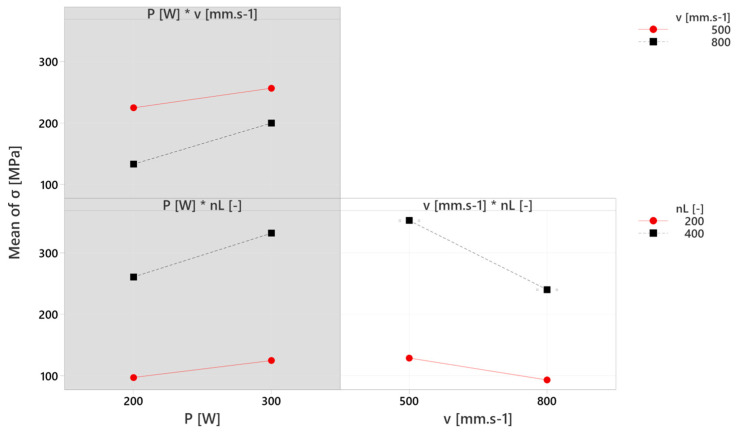
Interaction plot of factors (P, v and n_L_) effect on residual stresses (σ).

The interactions of individual factors and the influence of their combinations on the resulting residual stresses are included in Figure 12. The interaction plot for residual stresses σ [MPa] includes three partial graphs corresponding to each combination. The left upper graph includes the interaction of laser power (P) with scanning velocity (v) and it confirms, as previously described, the least influence of laser power on the increasing residual stresses, which can be restricted by increasing the scanning velocity (to 800 mm.s^−1^), where residual stresses can be decreased to 133.3 MPa. The lower graph on the left side includes the interaction of laser power (P) and the number of layers (n_L_), where the negative effect of the laser power is in this case supported by the increasing of layers, which is confirmed by the black dashed curve on the higher values of stresses and with the steeper slope. These two described interactions on the left side with the grey background were considered less significant and were not included in the creation of the regression model. The interaction of scanning velocity (v) and the number of layers (n_L_) was included in the creation of the regression model (white background) and its significance is confirmed by the steeper slope of the obtained curves, reaching higher values of residual stresses mainly in the case of the 400 layers. However, the curves are oriented in the direction of positive effect, due to the decrease of residual stresses with increasing scanning velocity.

**Figure 13:**
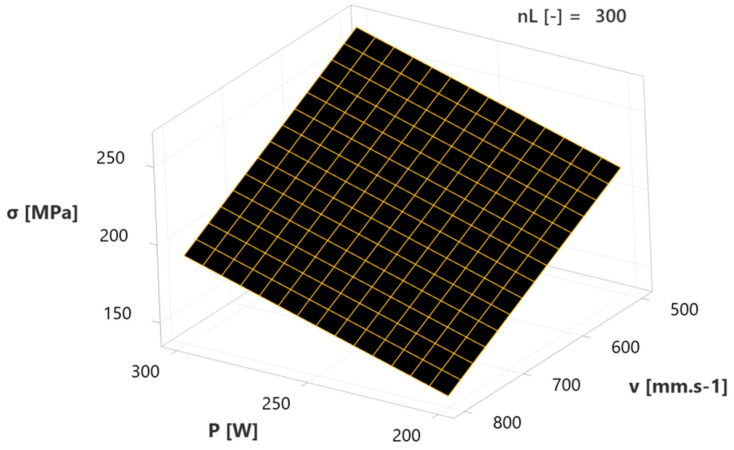
Surface plot of laser power—scanning velocity interaction influence on RS.

The following surface plots show the influence of two-factorial interactions (independent variables) on the response (dependent variable) in the form of residual stresses (σ), when the third factor is set to the mean value. The first diagram (Figure 13) depicts the combined influence of laser power (P) and scanning velocity (v) at 300 layers (n_L_). It is evident that a higher value of factor P causes an increased response (σ), which can be considered a negative influence. The maximum above 250 MPa was reached at 300 W and 500 mm.s^−1^. However, with the increase of factor v, which has a positive effect, a decrease of response (σ) occurs to a minimum of approx. 150 MPa at 200 W and 800 mm.s^−1^. It can be stated that increasing factor v leads to a decrease in negative factor P within the specified regression model.

**Figure 14:**
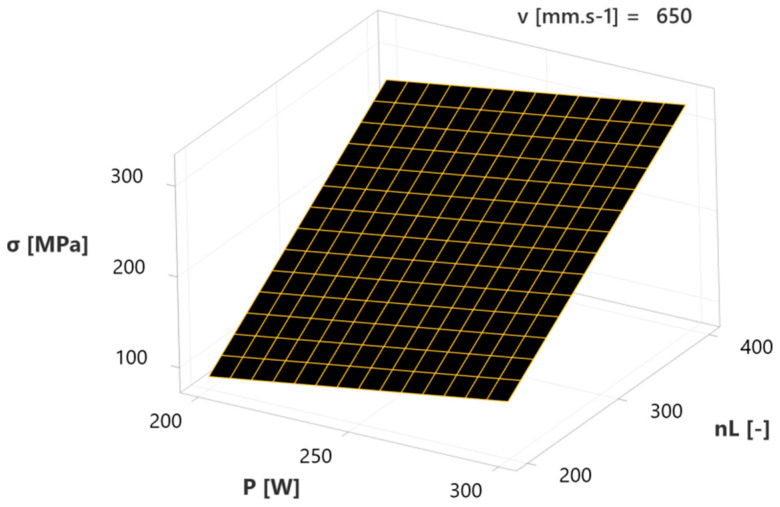
Surface plot of laser power—number of layers interaction influence on RS.

The next diagram (Figure 14) depicts a combination of the effect of laser power (P) and number of layers (n_L_) at a mean value of scanning velocity (v = 650 mm∙s^−1^). The maximum of residual stresses (approx. 300 W) is reached at a combination of 300 W with 400 layers and vice versa; the minimum value, approx. 100 MPa, is present at a combination of 200 W and 200 layers. Hence, the interaction of these two independent variables can be considered the negative effect of a specified regression model, due to the more significant induction of residual stresses (σ), caused by the increasing dependent variables’ values.

**Figure 15:**
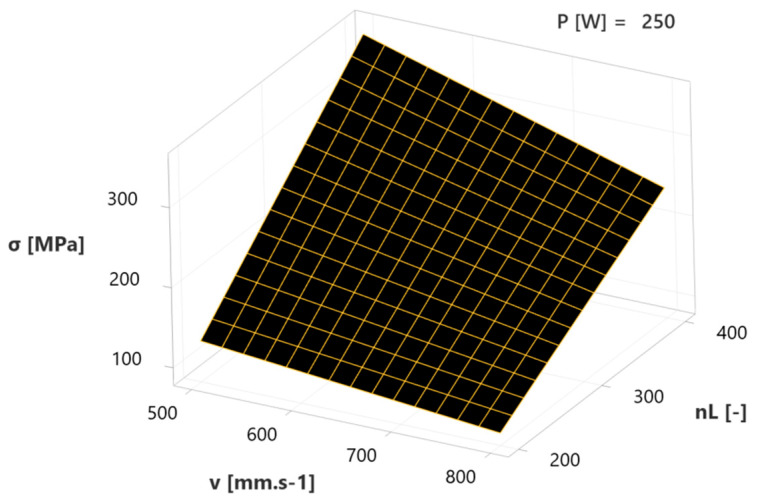
Surface plot of scanning velocity—number of layers interaction influence on RS.

The third diagram (Figure 15) includes the effects of the interaction of factors v and n_L_ at the mean value (250 W) of the third factor P. Similar to in a previous diagram (Figure 14), a dependence confirms the most significant effect of number of layers (n_L_) within the specified regression model, whereas in this case its influence can be restricted by the increase in positive effect values, in the form of scanning velocity (v), where at a combination of 200 layers and 800 mm.s^−1^, 100 MPa residual stresses are present. However, in the case of higher values of n_L_ (up to 400) and lower values of v (up to 500 mm.s^−1^), a considerable increase in response value (σ) occurs, with its maximum at 300 MPa.

## 4. Discussion

The obtained results of the described experiment are similar to the results of other studies in the field. The increase in residual stresses was recorded under the influence of setting the higher values of laser power (160 and 200 W) at the fixed scanning velocity (600 mm.s^−1^) within one study [8], which was confirmed by the described results, where higher values of residual stresses were measured at a higher laser power (Figure 11). During another experiment [6], the influence of overall energy density input was monitored, whereas its variability was reached by the various scanning velocities due to its more significant influence on residual stresses. It was confirmed within a study [5], where lower residual stresses were reached through increasing the scanning velocity (200, 400 and 800 mm.s^−1^) at a fixed laser power (200 W) and by the results interpreted in Figure 9 and Figure 13. However, FEM analysis with the experimental verification made by Waqar, Guo and Sun [18] points to the opposite consequence of increasing the scanning velocity. Similar to the case of increasing the laser power, the induction of residual stresses of higher values occurred. An older study [3] describes the number of applied layers as a significant residual stress factor. The mentioned parameter was shown to be the most influential within the described experiment (Figure 9 and Figure 11), and the only significant factorial interaction of the described regression model was reached by combining the number of layers with scanning velocity, whereas the first mentioned was more significant (Figure 15). Measured values of residual stresses within the experiment were similar to values stated within the referenced studies. Furthermore, we used a combination of statistical methods (DoE, ANOVA and regression analysis) provided the expression of individual and mutual dependencies of selected SLM parameters and their influence on RS in experimental stainless steel. A rarely considered factor in the form of the number of applied layers is shown to be important as individuals, so within interactions. Limits were set for the mentioned parameters in order to obtain a microstructure with the absence of significant voids (confirmed by SEM), which demonstrates the added value of the described experiment.

## 5. Conclusions

The described experiment includes results of the identification of residual stresses in a surface layer of SS 316L specimens made by SLM technology, measured using XRD. Specimens were manufactured using various combinations of technological parameters based on a DoE method, which subsequently served as input values for the statistical analysis of these factors on a residual stress state within the evaluated material. Based on experimental output data, the following conclusions can be derived:

- Promising application of Design of Experiments, ANOVA and regression analysis methods as suitable analytical tools for the evaluation of SLM technological parameters and their influence on final residual stresses in a surface layer of stainless steel.
- Proposal of regression equation based on the designed regression model, which could help to optimize the SLM process under similar conditions, considering the magnitude of induced residual stresses.
- The expression of correlations between selected technological parameters and induced residual stresses. A well-known negative effect of increasing laser power was confirmed and a considerably larger influence of a number of layers was presented. A positive effect of increasing scanning velocity was shown to be an effective tool for decreasing the negative effect of the other two factors.
- Effect on the residual stresses of mutual interactions between selected factors was described, with a significant influence of scanning velocity in combination with a number of layers.

Despite the promising obtained results, it is necessary to extend the presented research, which could help to optimize technology to a greater extent. Appropriate tools could include the application of more levels within individual factors (3-level model) and the use of center points. Increasing the replicates in the form of a higher number of experimental specimens could help to improve the credibility of the conducted regression analysis. A significant factor in the form of a number of applied layers seems to be obvious and necessary due to the dimensions of the final component. However, its influence and the influence of other factors are good to know, due to their prediction during the setting of appropriate parameters of post-processing operations.

## Figures and Tables

**Figure 1:**
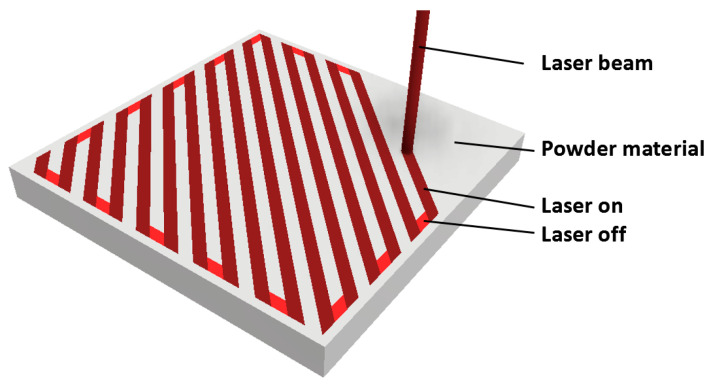
Meander scanning strategy used for SLM preparation of experimental specimens [28].

**Figure 3:**
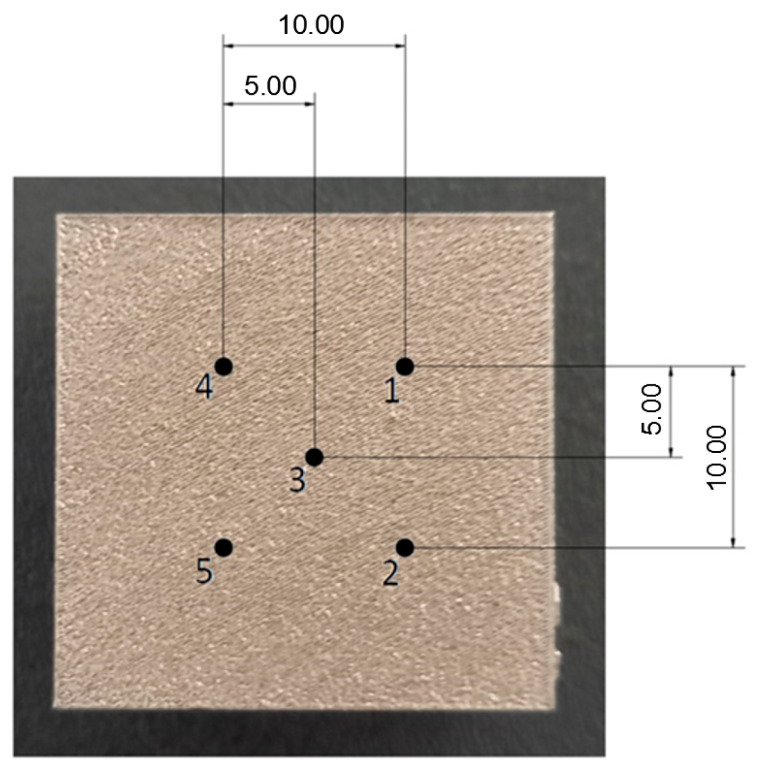
XRD measurement points on a surface layer of each specimen (dimensions in mm).

**Table 2:** Chemical composition of SS 316L stainless steel [26].

Element	Fe	Cr	Ni	Mo	Mn	Si	N	O	P	C	S
Mass (%)	Balance	16–18	10–14	2–3	≤2	≤1	≤0.1	≤0.1	≤0.045	≤0.03	≤0.03

**Table 3:** Physical properties of SS 316L stainless steel [26].

Parameter	Value	Unit
Density (wrought)	7.99	g∙cm^3^
Thermal conductivity	16.2	W/mK
Melting range	1371–1399	°C
Coefficient of thermal expansion	16∙10^−6^	K^−1^
Particle-size distribution	15–45	μm

**Table 5:** Combinations of technological parameters within specimens.

Specimen	P [W]	v [mm·s^−1^]	n_L_ [mm]
1	200	500	200
2	200	500	400
3	200	800	200
4	200	800	400
5	300	500	200
6	300	500	400
7	300	800	200
8	300	800	400
9	200	500	200
10	200	500	400
11	200	800	200
12	200	800	400
13	300	500	200
14	300	500	400
15	300	800	200
16	300	800	400

**Table 7:** Analysis of variance (ANOVA) for residual stresses.

Source	DF	Adj SS	Adj MS	F-Value	*p*-Value
Regression	4	174,885	43,721	67.74	0.000
Linear	3	168,986	56,329	87.27	0.000
P	1	9707	9707	15.04	0.003
v	1	21,938	21,938	33.99	0.000
n_L_	1	137,341	137,341	212.78	0.000
2-Way Interactions	1	5899	5899	9.14	0.012
v∙n_L_	1	5899	5899	9.14	0.012
Error	11	7100	645		
Lack-of-Fit	3	4597	1532	4.90	0.032
Pure Error	8	2503	313		
Total	15	181,985			

## Data Availability

Data are contained within the article.
